# Supplementary material for: Neuronal activity in sensory cortex predicts the specificity of learning in mice
Source: Nat Commun. 2022 Mar 4;13:1167. doi: 10.1038/s41467-022-28784-w (PMC8897443; doi:10.1038/s41467-022-28784-w)
Supplement: Supplementary file 1 — Supplementary Information [file 41467_2022_28784_MOESM1_ESM.pdf]

Supplementary Information (16 figures and 3 tables)

Supplementary Figure 1: Longitudinal two-photon imaging tracks activity of neurons over weeks.

Supplementary Figure 2: Anatomical location of imaging sites.

Supplementary Figure 3: Mean freezing to CS+ and CS- and learning specificity across sessions.

Supplementary Figure 4: Mean response to CS+ or CS- does not predict learning specificity

Supplementary Figure 5: Relationship between Zdiff and best frequency and anatomical location.

Supplementary Figure 6: Changes in neuronal discrimination between imaging sessions.

Supplementary Figure 7: Changes in frequency response after conditioning

Supplementary Figure 8: Changes in normalized responses and normalized responses post-DFC do not correlate with learning specificity.

Supplementary Figure 9: Changes in neuronal discriminability negatively correlate with learning specificity.

Supplementary Figure 10: Effects of best frequency distributions on changes in response post-DFC.

Supplementary Figure 11: Effects of region of sampling on changes in response post-DFC and prediction of learning specificity.

Supplementary Figure 12: Model schematic.

Supplementary Figure 13: Model schematic with PV inactivation of AC during DFC.

Supplementary Figure 14: Model schematic with AC inactivation during DFC.

Supplementary Figure 15: Model schematic with AC inactivation during memory recall.

Supplementary Figure 16: Auditory Brainstem Responses.

Supplementary Table 1: Statistics for all figures.

Supplementary Table 2: Statistics comparing normalized responses at CS+, CS- and CSc.

Supplementary Table 3: Statistics comparing non-normalized responses at CS+, CS- and CSc.

Supplementary Information

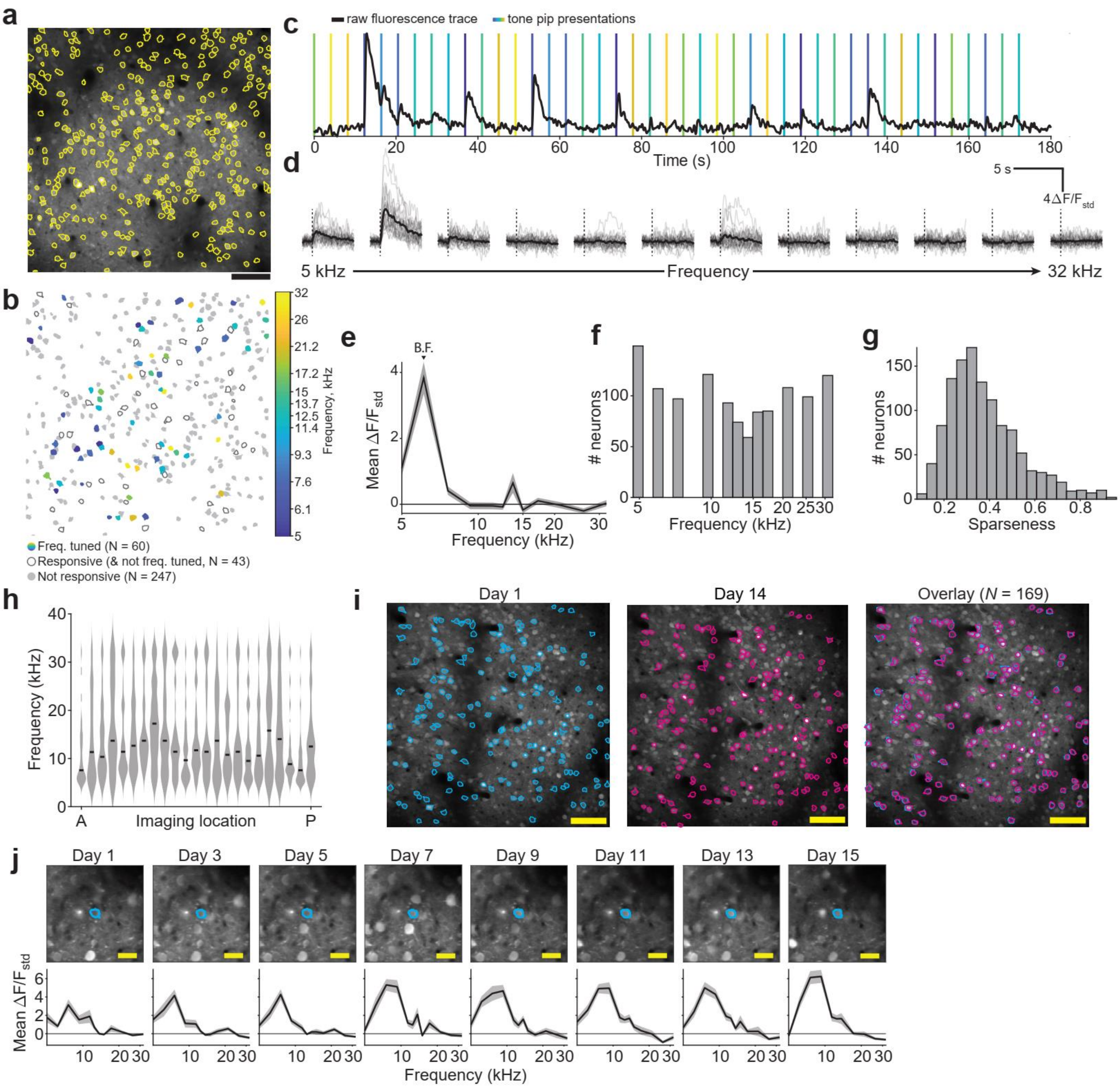

Supplementary Figure 1: Longitudinal two-photon imaging tracks activity of neurons over weeks. (a) Two-photon imaging field of view with regions of interest corresponding to individual neurons (yellow outline, N = 350). Scale bar = 100 µm. **(b)** Cell outlines from **a** indicating cells not responsive to the stimuli (light gray), cells responsive to tones (dark gray, t-test against zero, p < .05, corrected for multiple comparisons using Holm–Bonferroni method) but not frequency tuned and frequency-tuned cells (colored according to frequency tuning, significantly responsive and one-way ANOVA, p < .05). Color bar indicates best frequency of each tuned neuron. **(c)** Part of a raw fluorescence trace (black) for an example frequency-tuned neuron with tone pip presentation times overlaid in color (vertical lines). The color of the vertical lines corresponds to the frequency of the tone pip presented – colors as in **b**. **(d)** Responses of neuron in **c** with single-trial responses (gray, n = 25 for each frequency) and the mean response (black). Dashed lines = tone pip onsets. **(e)** Mean response (from tone onset to 2 s after tone onset) across trials at each frequency of neuron in **c-d**. This neuron has a best frequency (B.F.) of 6.1 kHz. **(f)** Distribution of best frequencies of responsive cells recorded 24 hours pre-DFC (N = 23 mice, n = 1148 neurons). **(g)** Distribution of sparseness of responsive cells recorded in **f**. **(h)** Smoothed best frequency distributions[1] of responsive neurons for each mouse pre-DFC ordered by anatomical location of the imaging window from anterior (A) to posterior (P). Black dashes indicate the median best frequency of all responsive neurons in the imaging window across sessions. **(i)** An example of neurons tracked between imaging sessions from one mouse. Field of view from two imaging sessions from the same mouse (maximum intensity projections), 15 days apart (left and middle) with ROIs tracked between the two sessions outlined in cyan and magenta. The right panel shows the ROIs from the two sessions overlaid. Scale bar = 100 µm. **(j)** Field of view and frequency responses of an example cell over the 8 sessions of the experiment. Cell is shown outlined (cyan line). Scale bar = 25 µm. Error bars = standard error of the mean (sem). Source data are provided as a Source Data file.

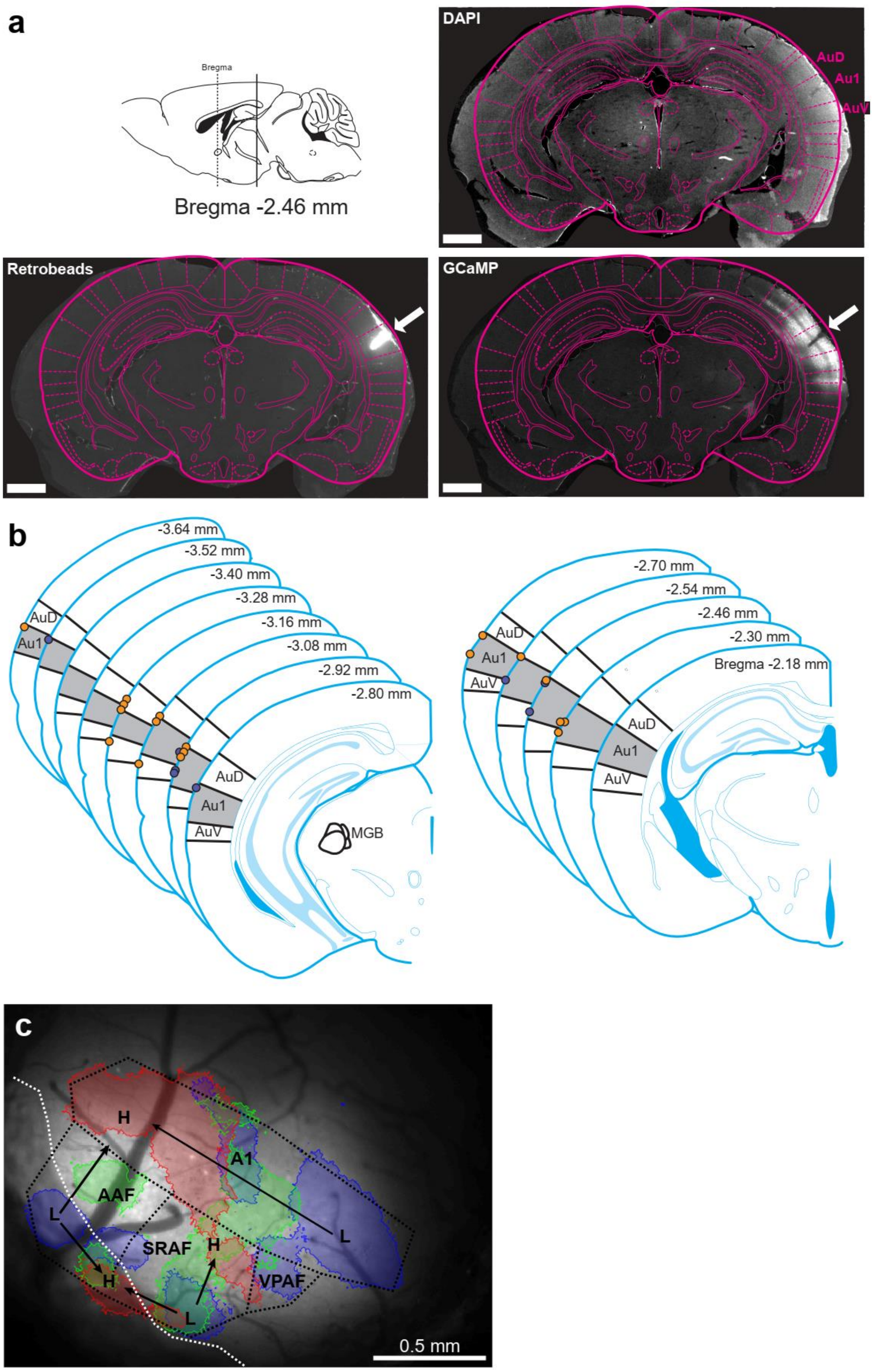

Supplementary Figure 2: Anatomical location of imaging sites. (a) A section from an example mouse is shown – this procedure was carried out on all mice and used to align the imaging site with the mouse brain atlas as in **b**. Upon completion of the experiments, the cranial window was removed, and a red indicator injected into the imaging site. The brains were fixed and sectioned at 40µm. We applied DAPI to reveal cell nuclei. This stain was used to align the section and the red indicator injection site (white arrows) with the mouse brain atlas (magenta outline). The red channel reveals the site of injection (Retrobeads) and is aligned with strong GCaMP6m/s expression (GCaMP). Scale bar = 1 mm. **(b)** Using the red injection sites as markers for the location of the imaging windows, the brain sections were aligned with the mouse brain atlas[2] to obtain the anterior-posterior location of the imaging windows relative to bregma and the field of auditory cortex imaged from. The center of the imaging field of view on the surface of the brain of conditioned (yellow) and pseudo-conditioned (blue) mice (N = 26/28) is indicated on the mouse brain atlas adapted from. The left stack of sections contains the auditory thalamus (medial geniculate body, MGB), and the more anterior right-hand stack of sections does not contain the MGB. **(c)** For two mice, the red injection failed, and we used widefield imaging to locate the field of view. The figure shows an example widefield result. Thresholded responses to low (5 kHz, blue), medium (15 kHz, green) and high (30 kHz, red) tones of each pixel are indicated by the shaded regions. The pattern of responses allowed us to estimate the locations of the auditory fields (black dashed lines) using Romero et al.[3] as a guide. A1 – primary auditory cortex, VPAF – ventral posterior auditory field, SRAF – suprarhinal auditory field, AAF – anterior auditory field. Arrows indicate tonotopic gradients from low (L) to high (H) frequency. White-dotted line indicates dental cement at the edge of the imaging window. Source data are provided as a Source Data file.

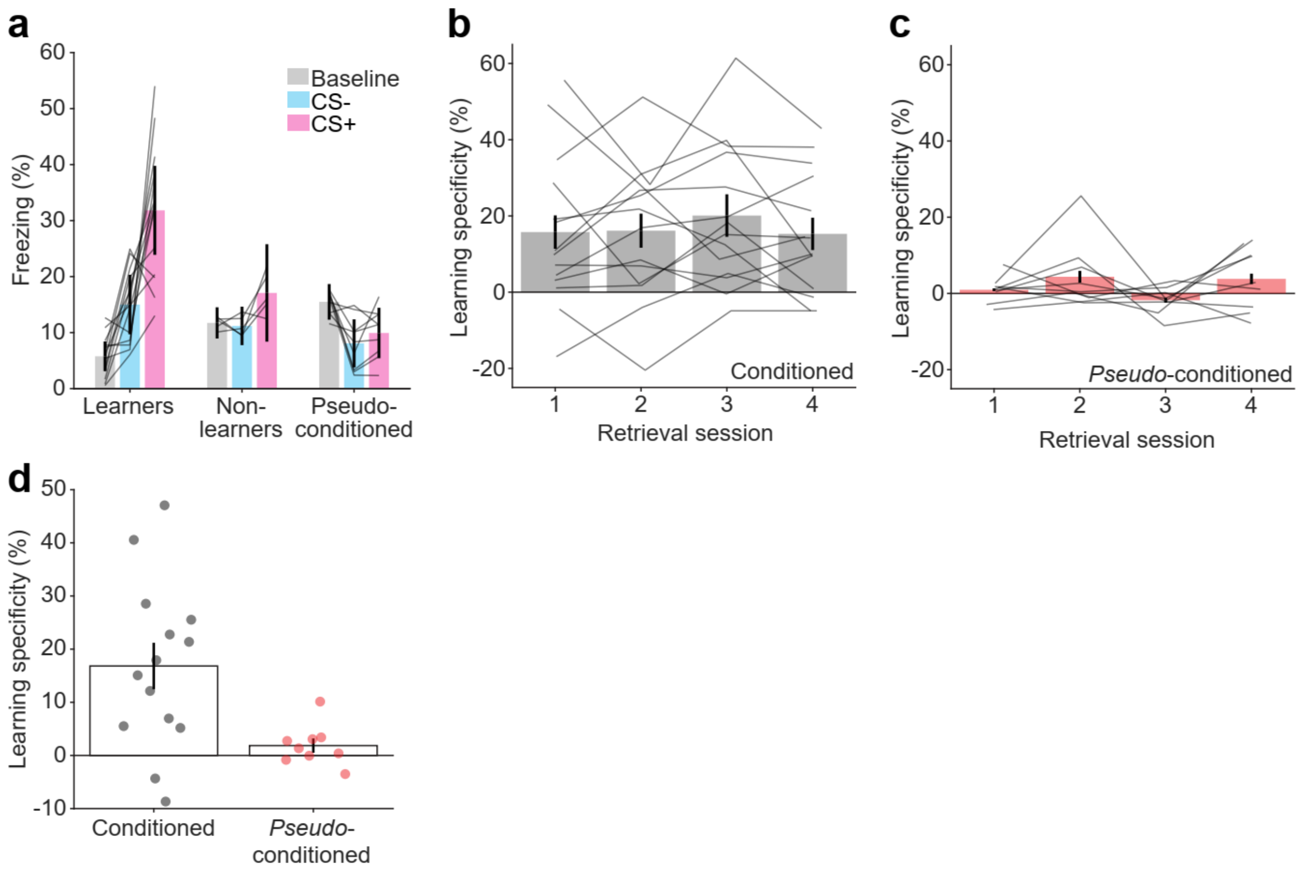

Supplementary Figure 3: Mean freezing to CS+ and CS- and learning specificity across sessions. (a) Mean (± sem) freezing across all 4 retrieval sessions at baseline (gray) and in response to CS+ (pink) and CS- (blue). Learners (*N* = 14) showed a significant difference from baseline freezing (baseline vs non-baseline) while non-learners (*N* = 5) did not (see Methods). Non-learners were subsequently excluded from further analysis. *Pseudo*-conditioned mice *N* = 9. Gray lines indicate individual mice. **(b)** Mean (± sem) learning specificity in each retrieval session for conditioned mice (*N* = 14). Gray lines show individual mice. **(c)** Same as **b** for *pseudo*-conditioned mice (*N* = 9). **(d)** Mean (± sem) learning specificity across all 4 retrieval sessions for conditioned (gray, *N* = 14) and *pseudo*-conditioned mice (red, *N* = 9). Circles show individual mice. Source data are provided as a Source Data file.

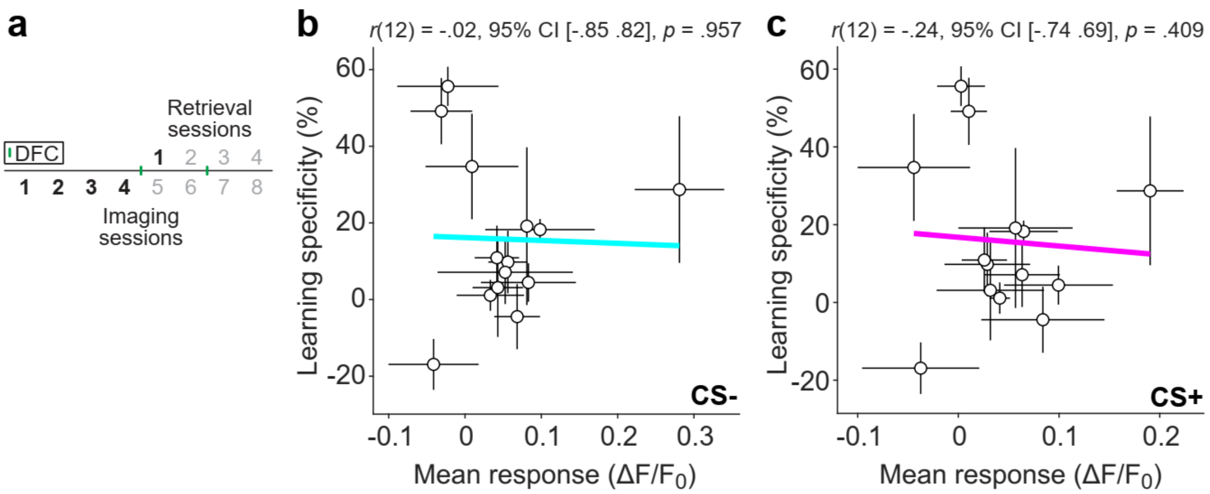

Supplementary Figure 4: Mean response to CS+ or CS- does not predict learning specificity. (a) Responses to CS+ and CS- of responsive cells were averaged in each session and across the 4 pre-DFC imaging sessions (1-4). These mean responses were compared with the learning specificity from retrieval session 1 (24 hours post-DFC). **(b)** Mean (± sem) learning specificity of conditioned mice (*N* = 14) as a function of the mean (± sem) response to CS- pre-DFC. The line shows the linear best fit. Error bars represent standard error of the mean. Statistics: two-tailed Spearman's rank correlation. **(c)** Same as **b** but for responses to CS+. Statistics: two-tailed Spearman's rank correlation. Source data are provided as a Source Data file.

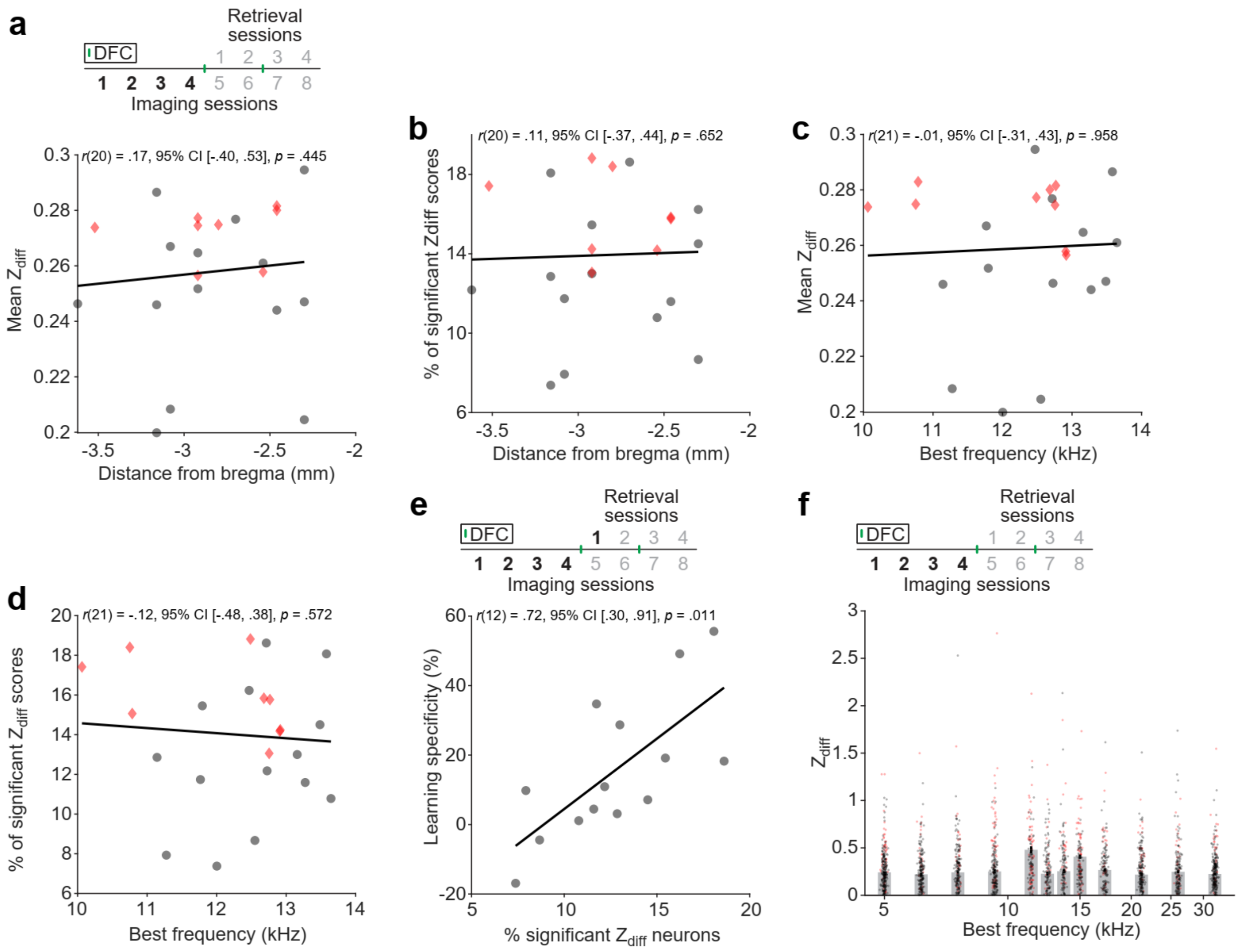

Supplementary Figure 5: Relationship between Z_{diff} and best frequency and anatomical location. (a) Mean Z_{diff} of responsive neurons was averaged in each session and across sessions for each mouse. Relationship between the distance from bregma of the imaging field of view and the mean Z_{diff} scores of conditioned (black circles) and *pseudo*-conditioned mice (red diamonds) across pre-DFC sessions. **(b)** Relationship between the distance from bregma of the imaging field of view and the % of significant Z_{diff} scores across pre-DFC sessions. **(c)** Relationship between mean best frequency of responsive units in the imaging field of view and the mean Z_{diff} scores across pre-DFC sessions. **(d)** Relationship between mean best frequency of responsive units in the imaging field of view and the % of significant Z_{diff} scores across pre-DFC sessions**. (e)** Relationship between the mean % of significant Z_{diff} scores in the imaging field of view across pre-DFC sessions and the learning specificity from retrieval session 1 for conditioned mice. **(f)** Relationship between mean Z_{diff} across pre-DFC sessions for each neuron and best frequency. Grey bars show median Z_{diff} ± sem. Statistics **a** - **e**: two-tailed Spearman's rank correlation. Source data are provided as a Source Data file.

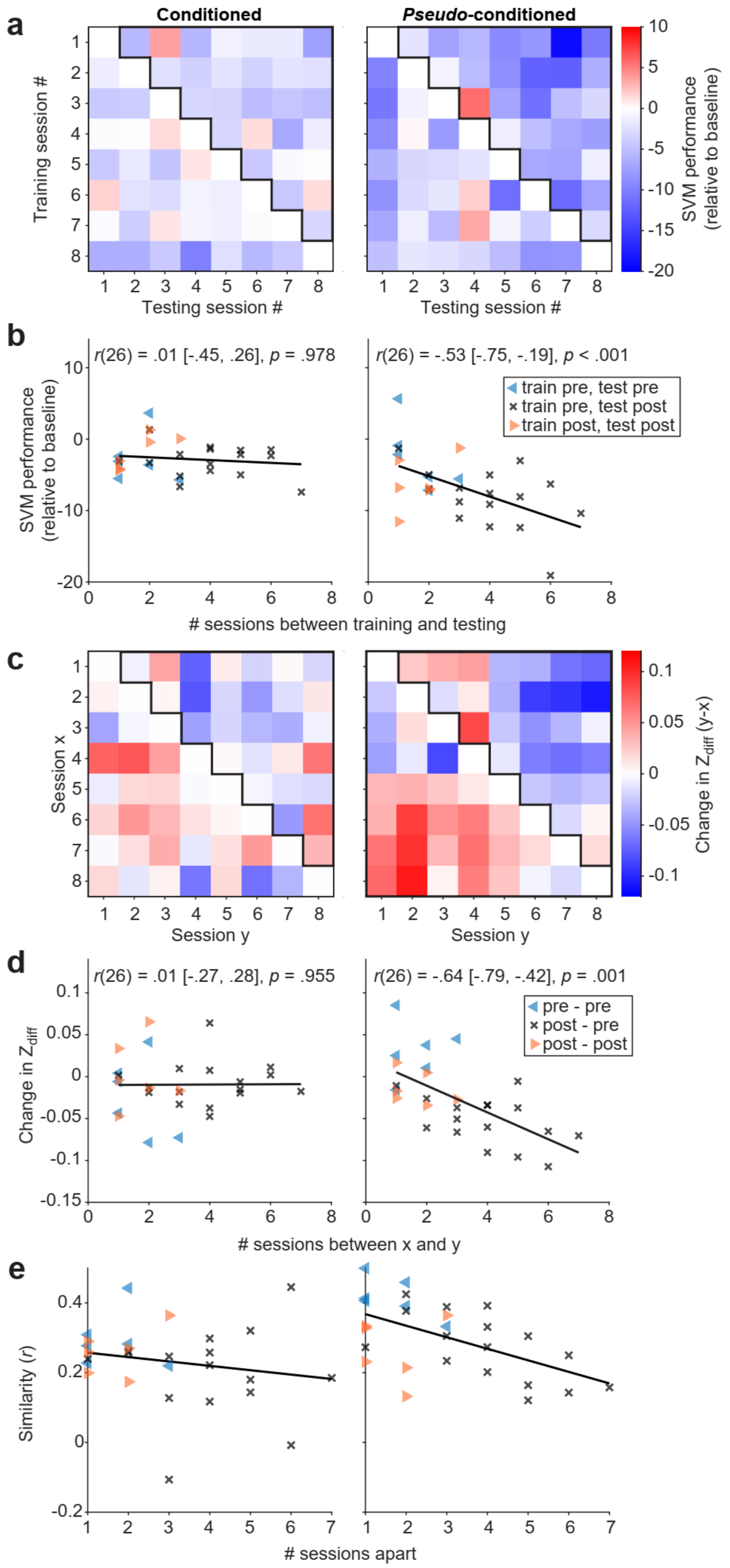

Supplementary Figure 6: Changes in neuronal discrimination between imaging sessions. (a) The SVM was trained (with 10-fold cross validation) with data from a single imaging session (training session) and subsequently tested on data from the same neurons recorded from a second imaging session (testing session). Since the number of neurons tracked between each pair of sessions varied, performance of the SVM on the testing data is shown relative to the baseline of performance of the SVM on data left out from the training session. SVM performance is shown averaged across mice. **(b)** The relationship between the difference in performance from **a** and the number of sessions between each pair in the forward time-direction (upper triangle outlined in **a**). Black lines show best linear fit. Statistics: two-tailed Spearman's rank correlation *r* [95% CI]. **(c)** The difference in Z_{diff} between neurons tracked between pairs of imaging sessions for conditioned and *pseudo*-conditioned mice. **(d)** The relationship between difference in Z_{diff} and number of sessions between each pair in the forward direction (upper triangle outlined in **c**). Statistics: two-tailed Spearman's rank correlation *r* [95% CI]. **(e)** Similarity of the Z_{diff} scores of neurons tracked between pairs of sessions were assessed using Pearson's correlation (*r*). The panels show the similarity between each pair of sessions averaged across conditioned (left) and *pseudo*-conditioned mice (right). Black lines show best linear fit. Source data are provided as a Source Data file.

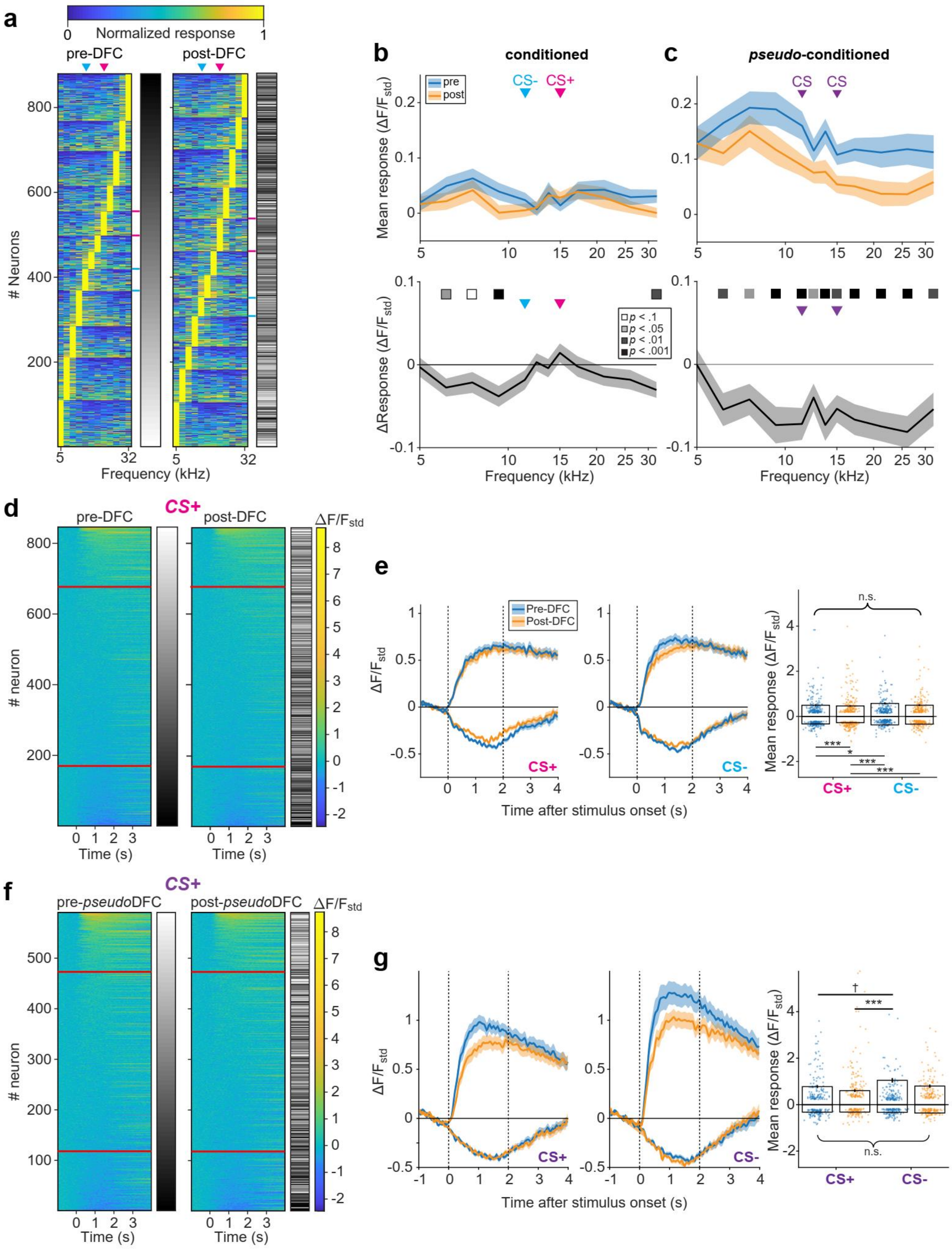

Supplementary Figure 7: Changes in frequency response after conditioning. (a) Normalized frequency response curves of all neurons responsive at least once pre-DFC (imaging sessions 1-4) and once post-DFC (imaging sessions 5-8). Responses from neurons present in more than one session pre- or post-DFC are averaged together. Neurons are ordered according to their best frequency and secondarily by sparseness. The grey bars indicate the identity of each neuron pre- and post-DFC. The bars indicate the neurons with CS- (cyan) and CS+ (magenta) best frequencies. **(b)** (Upper panel) The mean (± sem) frequency response curve of all responsive neurons (*not* normalized) pre- and post-DFC. (Lower panel) Mean (± sem) change in frequency response curve across responsive all neurons. **(c)** Same as **b** for *pseudo*-conditioned mice. Statistics: two-way rm-ANOVA, Tukey-Kramer post-hoc analysis, Table S1. **(d)** Mean CS+-responses ordered by magnitude from conditioned mice (N = 14 mice) pre-DFC (left) and post-DFC (right). The grey bars indicate the identity of each neuron pre- and post-DFC. Red lines indicate the upper and lower 20th percentiles (N = 176 neurons). **(e)** Mean (± sem) responses to CS+ (left panel) and CS- (middle panel) of the upper and lower percentile of neurons in **d**. Right panel: Mean (± sem) positive and negative responses from stimulus onset to 2 seconds after onset (dotted lines in left and middle panels) to CS+ and CS- pre- and post-DFC. Dots show individual neurons. Statistics: 2-way ANOVA with Tukey's multiple comparisons test, Table S1. **(f)** Same as **d** but for responses to CS+ from *pseudo*-conditioned mice (N = 9 mice, N = 126 neurons). **(g)** Same as **e** but for responses to the CS+ and CS- in *pseudo*-conditioned mice. Statistics for **e** and **g**: Data are shown as mean ± sem. $^{\dagger}p < 0.1$, $^{*}p < 0.05$, $^{**}p < 0.01$, $^{***}p < 0.001$, $^{n.s.}p > 0.10$. Source data are provided as a Source Data file.

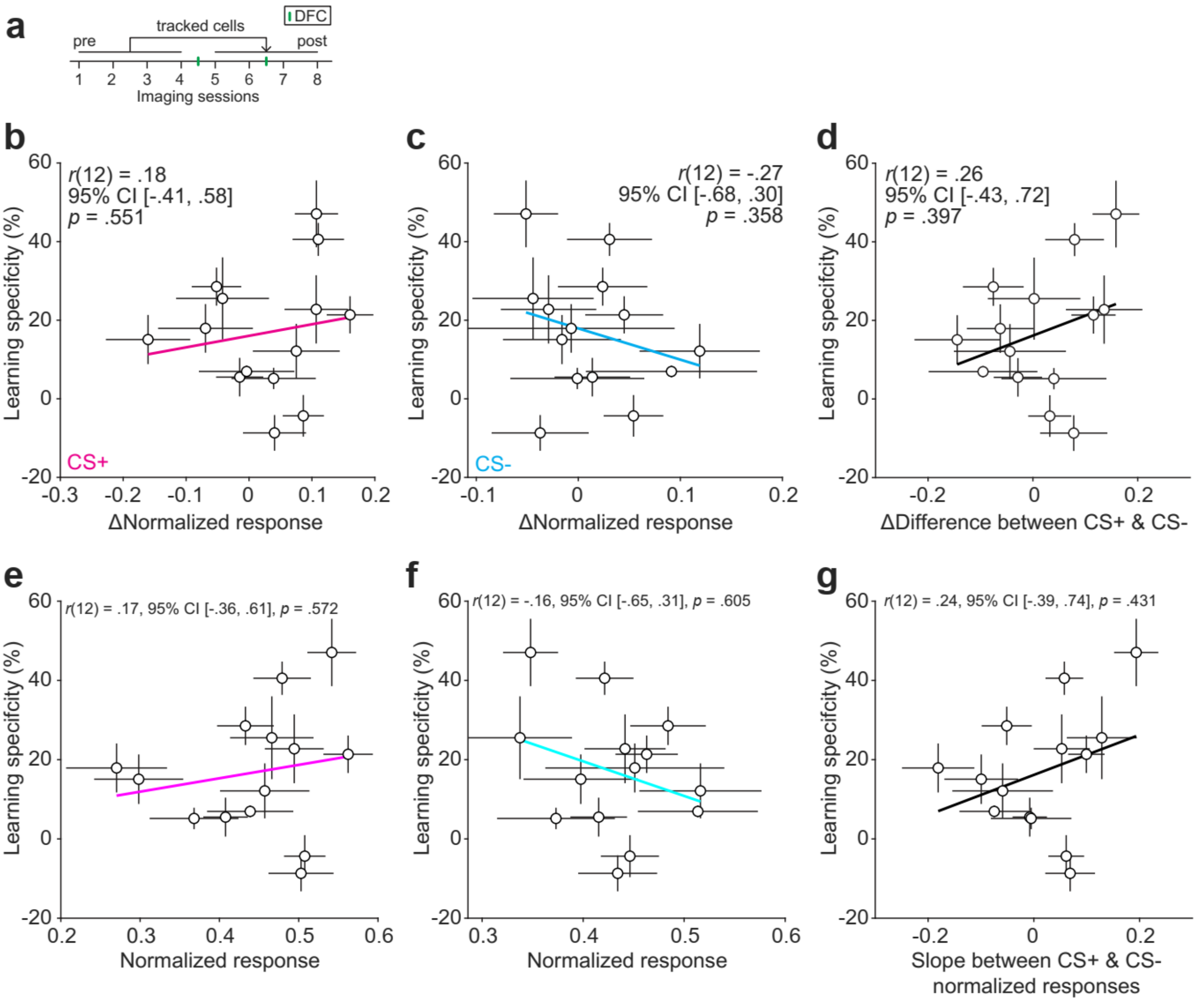

Supplementary Figure 8: Changes in normalized responses and normalized responses post-DFC do not correlate with learning specificity. (a) Cells included were responsive in at least one imaging session pre- (sessions 1-4) and post-DFC (sessions 5-8). **(b)** Mean learning specificity across retrieval sessions 1-4 as a function of change in normalized response magnitude to CS+ post-DFC. Magenta line represents the best linear fit. **(c)** Same as **b** but for change in response to CS-. **(d)** Same as **b** but for change in difference between CS+ and CS-. **(e)** Mean learning specificity across retrieval sessions 1-4 as a function of normalized response magnitude to CS+ post-DFC. Magenta line represents the best linear fit. **(f)** Same as **e** but for response to CS-. **(g)** Same as **e** but for difference in normalized response magnitude between CS+ and CS-. Statistics **b** - **g**: two-tailed Spearman's rank correlation (*N* = 14). Data are shown as mean ± sem. Source data are provided as a Source Data file.

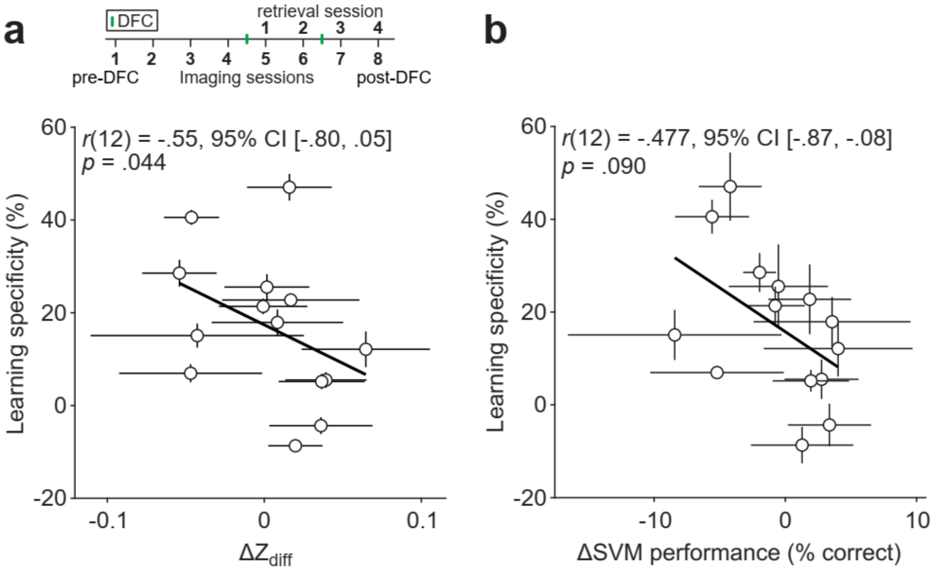

Supplementary Figure 9: Changes in neuronal discriminability negatively correlate with learning specificity. (a) Change in neuronal discriminability was calculated as the difference between the mean discriminability across pre-DFC imaging sessions (1-4) and the mean discriminability across post-DFC imaging sessions (5-8). Learning specificity post-DFC (retrieval sessions 1-4) as a function of change in Z_{diff} from pre- to post-DFC. Black line represents the best linear fit. **(b)** Same as **a** but for change in SVM performance. Data are shown as mean ± sem. Statistics: two-tailed Spearman's rank correlation. $^{\dagger}p < 0.1$, $^{*}p < .05$, $^{**}p < .01$, $^{***}p < 0.001$, $^{n.s.}p > 0.10$. Source data are provided as a Source Data file.

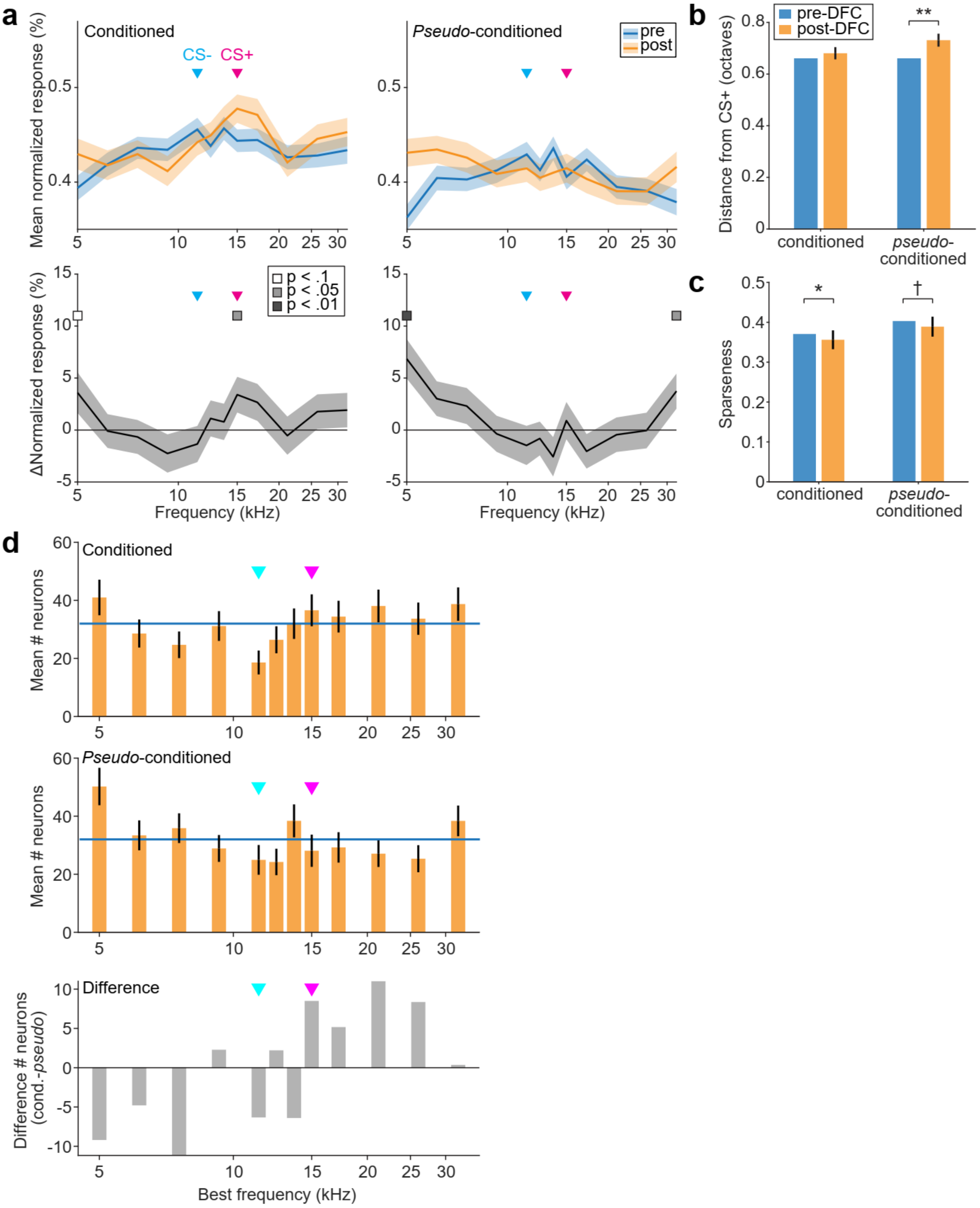

Supplementary Figure 10: Effects of best frequency distributions on changes in response post-DFC. (a) The minimum number of neurons with best frequency at each tested frequency pre-DFC (n = 32) across conditioned and *pseudo*-conditioned mice was resampled with replacement (x250) from populations of neurons with best frequency at each frequency in conditioned and *pseudo*-conditioned mice. This had the effect to normalize the pre-DFC frequency distributions across neurons from the conditioned and *pseudo*-conditioned mice. The top panels show the mean (± sd) normalized responses pre- (blue) and post-DFC (orange) for conditioned and *pseudo*-conditioned mice. The bottom panels show the mean (± sd) % change in normalized response for the two groups. **(b)** Mean (± sd) distance of the best frequency from CS+ pre- and post-DFC, using the same resampling as in **a**, n = 250 resamples. **(c)** Mean (± sd) sparseness of frequency tuning pre- and post-DFC, using the same resampling as in **a**, n = 250 resamples. **(d)** Mean (± sd) best frequency distributions post-DFC of the resampled neurons from **a** for conditioned (top, N = 14 mice) and *pseudo*-conditioned (middle, N = 9 mice) mice, n = 250 resamples. The pre-DFC distribution is indicated by the blue line. The bottom panel shows the difference between the post-DFC best frequency distributions of the conditioned and *pseudo*-conditioned mice. Statistics **a** – **c**: Significance p-values in indicate the percentile of the shuffled distributions at which zero occurred for the difference between the pre- and post-DFC for each frequency (**a**), distance of best frequency from CS+ (**b**) and sparseness (**c**). Error bars in all panels: ± standard deviation of resampled data. $^{\dagger}p < 0.1$, $^{*}p < .05$, $^{**}p < .01$, $^{***}p < 0.001$, $^{n.s.}p > 0.10$. Source data are provided as a Source Data file.

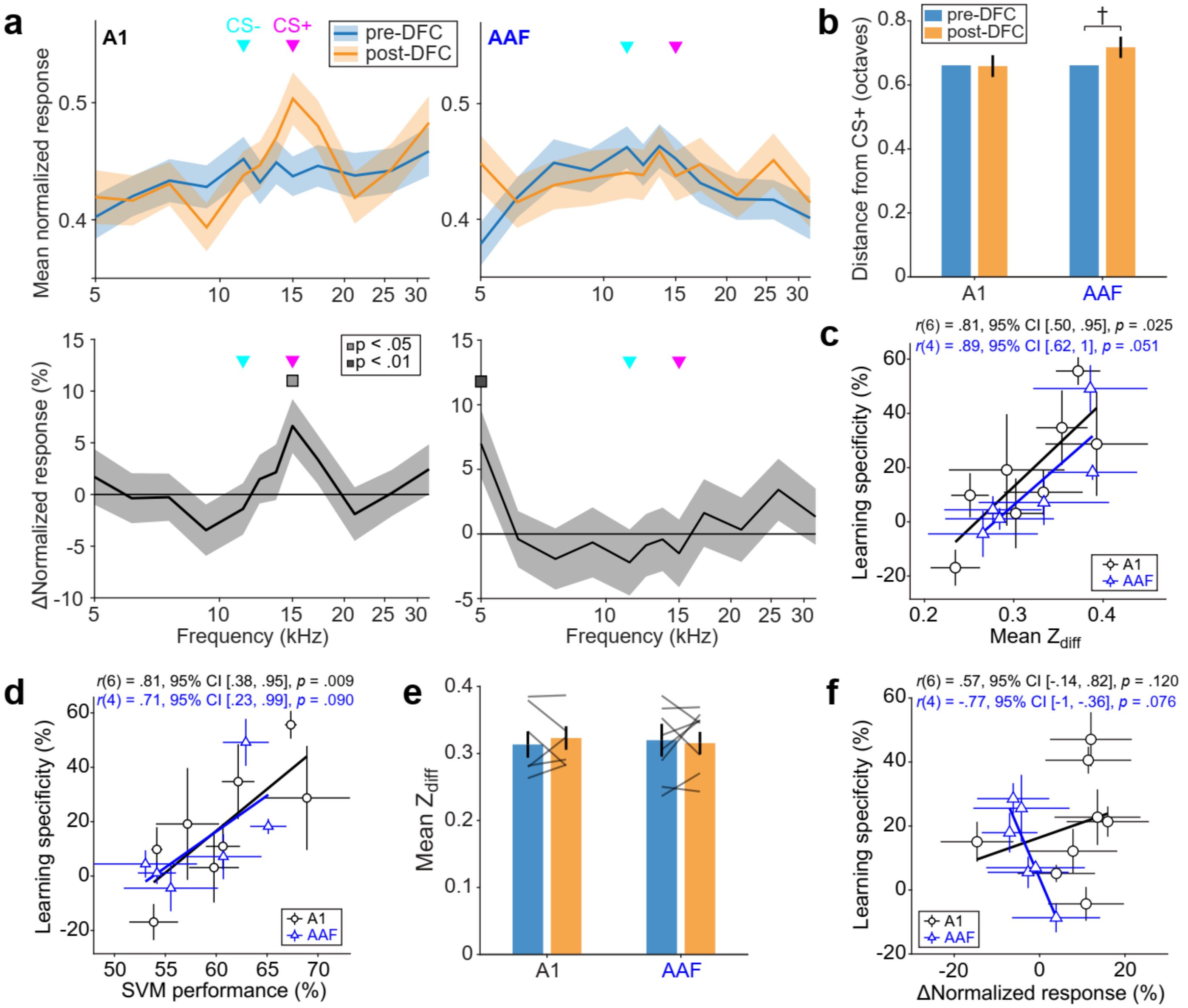

Supplementary Figure 11: Effects of region of sampling on changes in response post-DFC and prediction of learning specificity. (a) The minimum number of neurons with best frequency at each tested frequency pre-DFC (n = 16) across conditioned mice with imaging regions in putative A1 or AAF was resampled with replacement (x250) from populations of neurons with best frequency at each frequency. This had the effect to normalize the pre-DFC frequency distributions across neurons from imaging windows in A1 or AAF. The top panels show the mean (± sd) normalized responses pre- (blue) and post-DFC (orange) for A1 and AAF in conditioned mice. The bottom panels show the mean (± sd) % change in normalized response for the two groups. Significance p-values indicate the percentile of the shuffled distributions at which zero occurred for the difference between the pre- and post-DFC for each frequency **(b)** Mean (± sd) distance of the best frequency from CS+ pre- and post-DFC, using the same resampling as in **a**, n = 250 resamples. Significance p-values indicate the percentile of the shuffled distributions at which zero occurred for the difference between the pre- and post-DFC distance of best frequency from CS+**. (c)** Relationship between mean Z_{diff} (± sd) across pre-DFC imaging sessions (1-4) and learning specificity (± sem) in retrieval session 1 for conditioned mice (N = 14) with imaging regions in putative A1 (black) and AAF (blue). Statistics: two-tailed Spearman's rank correlation. **(d)** Relationship between mean (± sem) SVM performance across pre-DFC imaging sessions (1-4) and learning specificity (± sem) in retrieval session 1 for conditioned mice with imaging regions in putative A1 (black) and AAF (blue). Statistics: two-tailed Spearman's rank correlation. **(e)** Change in mean Z_{diff} (± sem) from pre- to post-DFC for conditioned mice (N = 14) with imaging regions in A1 (N = 8 mice) and AAF (N = 6 mice). **(f)** Relationship between mean change in normalized response at CS+ (± sd) of neurons tracked from pre- to post-DFC and mean learning specificity (± sem) post-DFC (retrieval sessions 1-4) across mice. Statistics: two-tailed Spearman's rank correlation. Error bars in **a** and **b**: ± standard deviation of resampled data from **a**. $^{\dagger}p < 0.1$, $^{*}p < .05$, $^{**}p < .01$, $^{***}p < 0.001$, $^{n.s.}p > 0.10$. Source data are provided as a Source Data file.

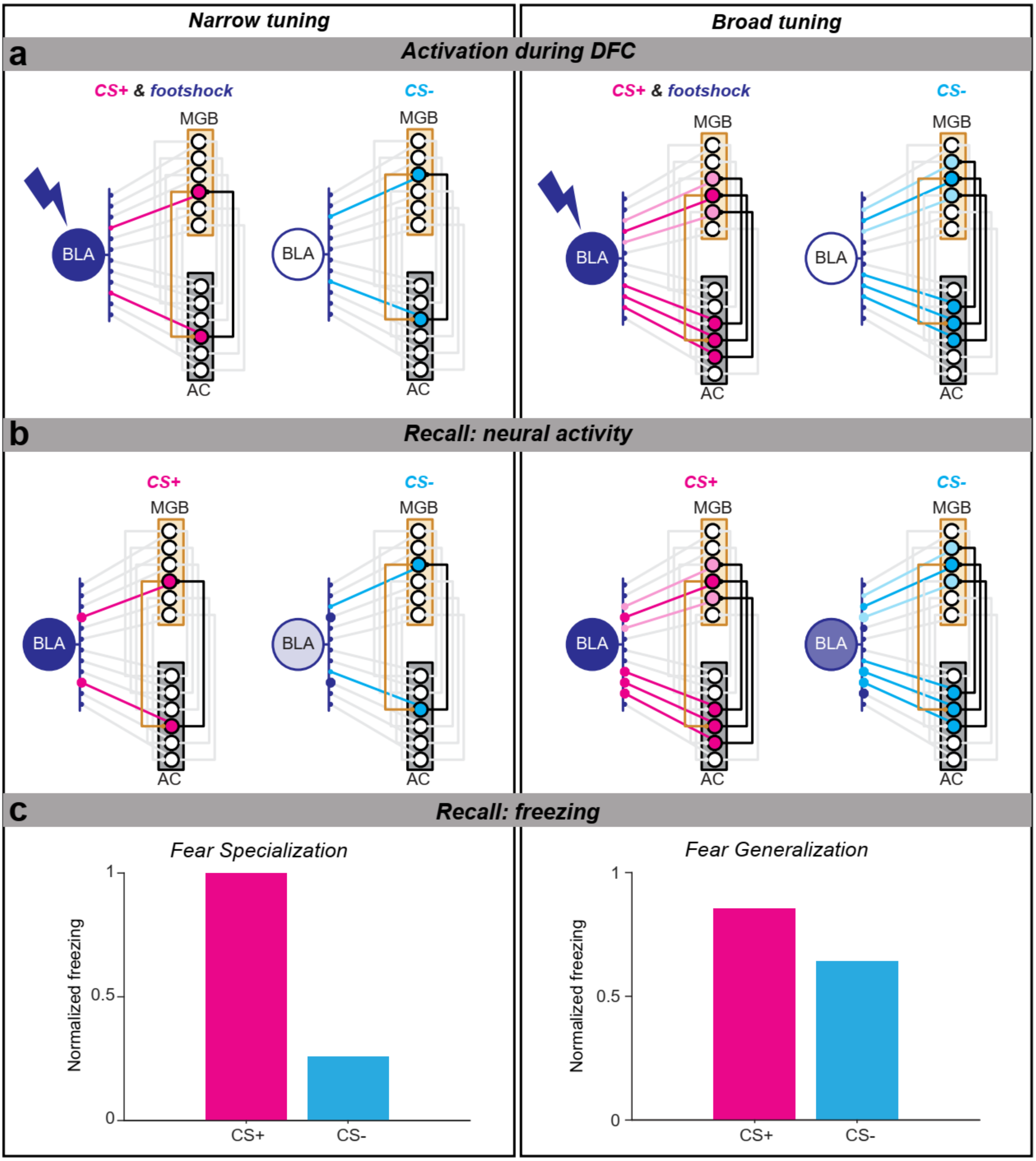

Supplementary Figure 12: Model schematic. The stages of the model are outlined with narrow tuning in AC (high discriminability, left column) and broad tuning (low discriminability, right column). **(a)** Activation of projections during DFC. MGB is narrowly tuned and provides input to AC (orange connections). Feedback from AC to MGB (black connections) is narrow or broad depending upon the tuning width of AC, e.g. in different subjects. A foot-shock (lightning shape) is delivered simultaneously with the CS+ (magenta) and activates the BLA. CS- (cyan) is presented alone and there is no activation of BLA. The weights of the connections between AC and BLA, and MGB and BLA are strengthened depending on their co-activation of BLA. Feedback connections from AC help to strengthen the MGB projections to BLA. Connections with increased weights are shown as larger circles. **(b)** During memory recall, with narrow AC tuning, CS+ stimulus activates consolidated connections, thus activating BLA, whereas the CS- does not. With broad AC tuning, both CS+ and CS- activate consolidated connections, leading to BLA activation with both stimuli. **(c)** The normalized freezing output of the model (relative levels of BLA activation). Broad tuning leads to increased levels of fear generalization. Source data are provided as a Source Data file.

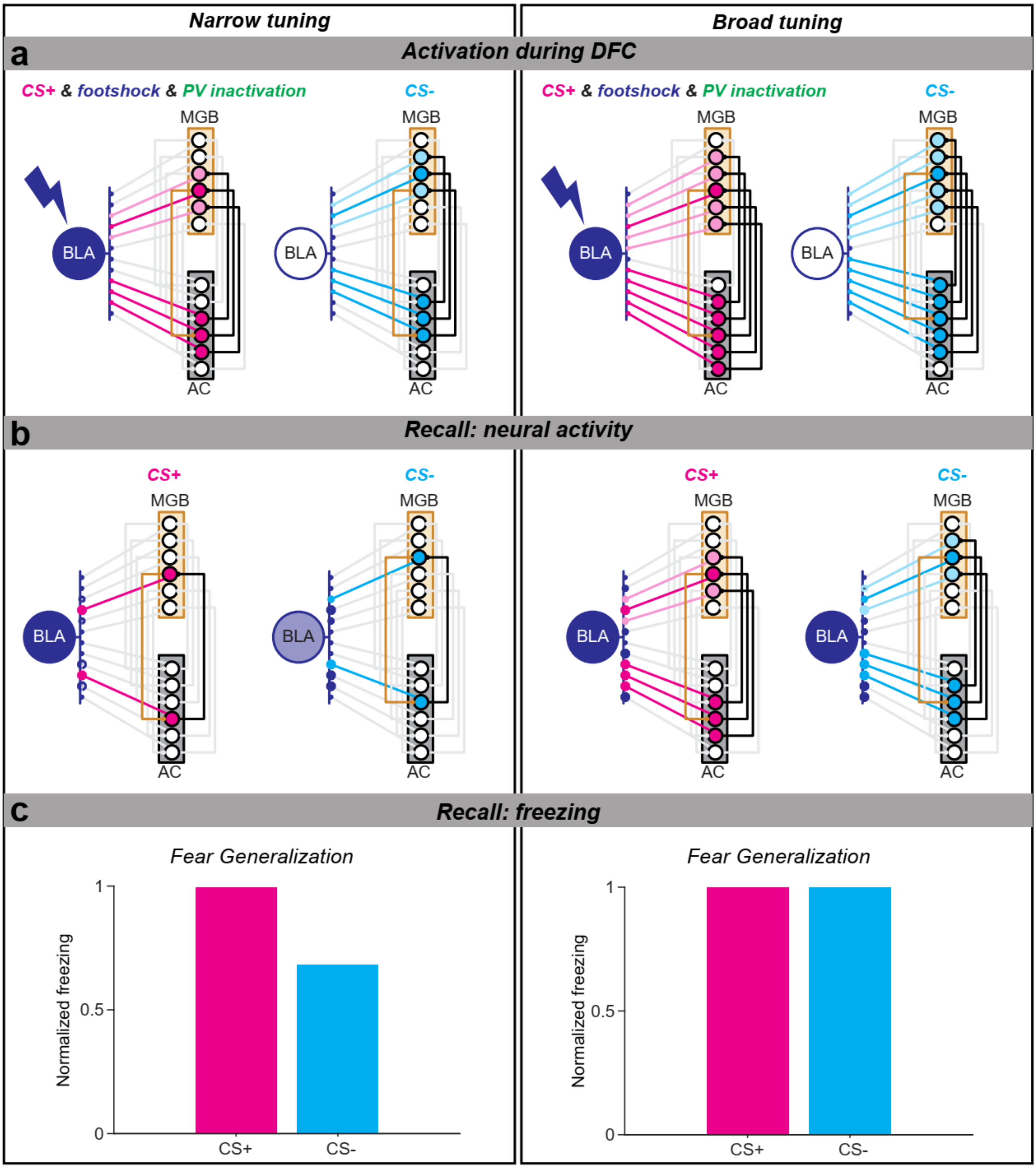

Supplementary Figure 13: Model schematic with PV inactivation of AC during DFC. The process of the model is the same as Supplementary Fig. 12. PV inactivation is modeled by lowering cortical inhibition. It effectively increases the tuning width of AC tuning (therefore lower discrimination in AC). Following activation (**a**), AC tuning is returned to its original narrow and broad tuning (as in Supplementary Fig. 11). Even with narrow tuning, CS- now activates strengthened AC-BLA and MGB-BLA connections (**b,** left panel) leading to increased fear generalization (**C,** left panel) compared with no PV inactivation (Supplementary Fig. 12). With broad tuning, both CS+ and CS- activate many strengthened AC-BLA and MGB-BLA connections (**b**, right panel) leading to high levels of fear generalization compared with no PV inactivation (**c**, right panel). Source data are provided as a Source Data file.

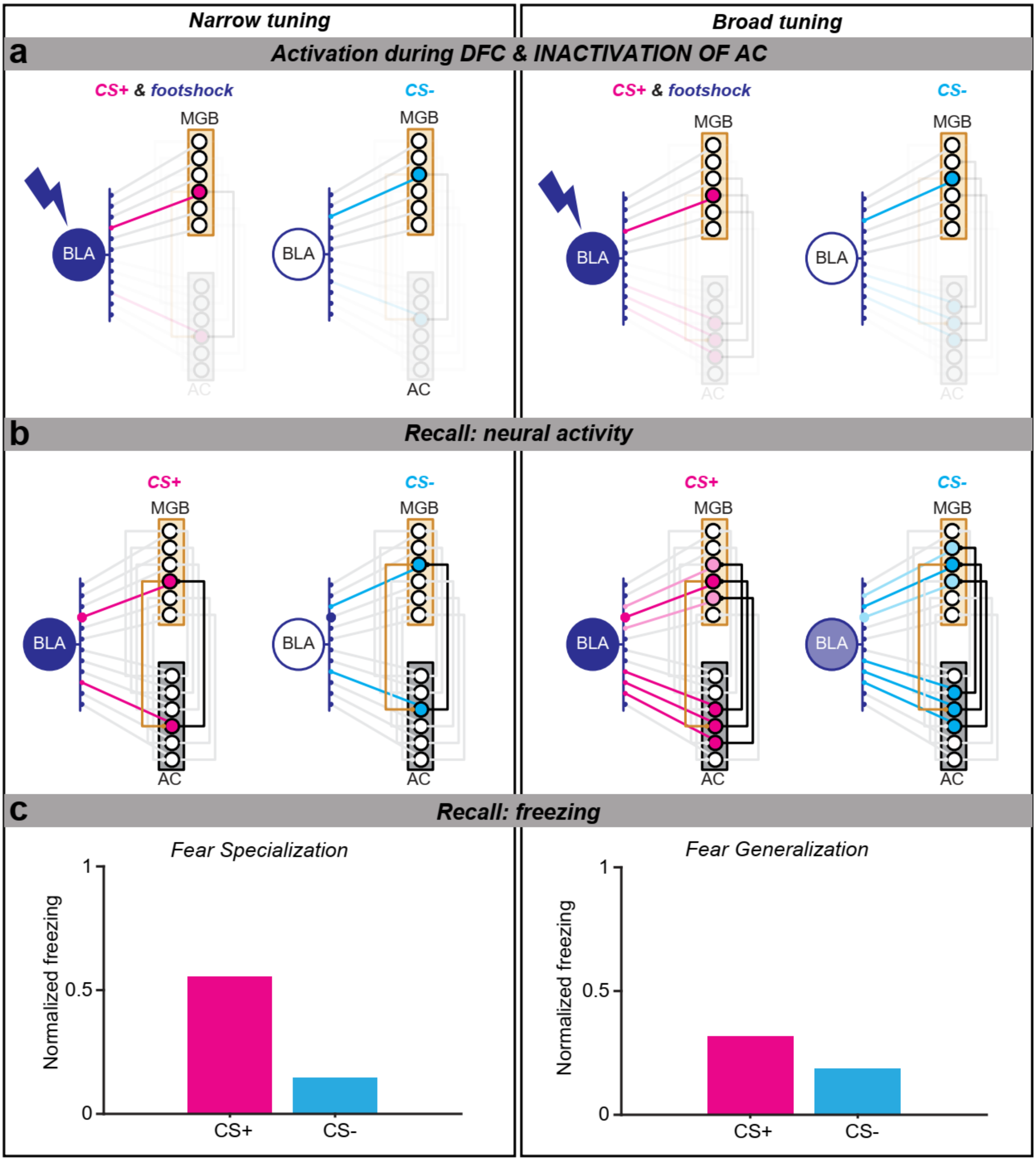

Supplementary Figure 14: Model schematic with AC inactivation during DFC. The process of the model is the same as Supplementary Fig. 12 AC inactivation is modeled by setting cortical currents to zero during the conditioning phase. Following inactivation (**a**), AC tuning is returned to its original narrow and broad tuning (as in Supplementary Fig. 12). **(b)** Schematic of recall with AC active. **(c)** Freezing to CS+ and CS- presentations during recall. Although freezing is lower in general, there is still discrimination in both cases but much lower than when AC is active throughout. Source data are provided as a Source Data file.

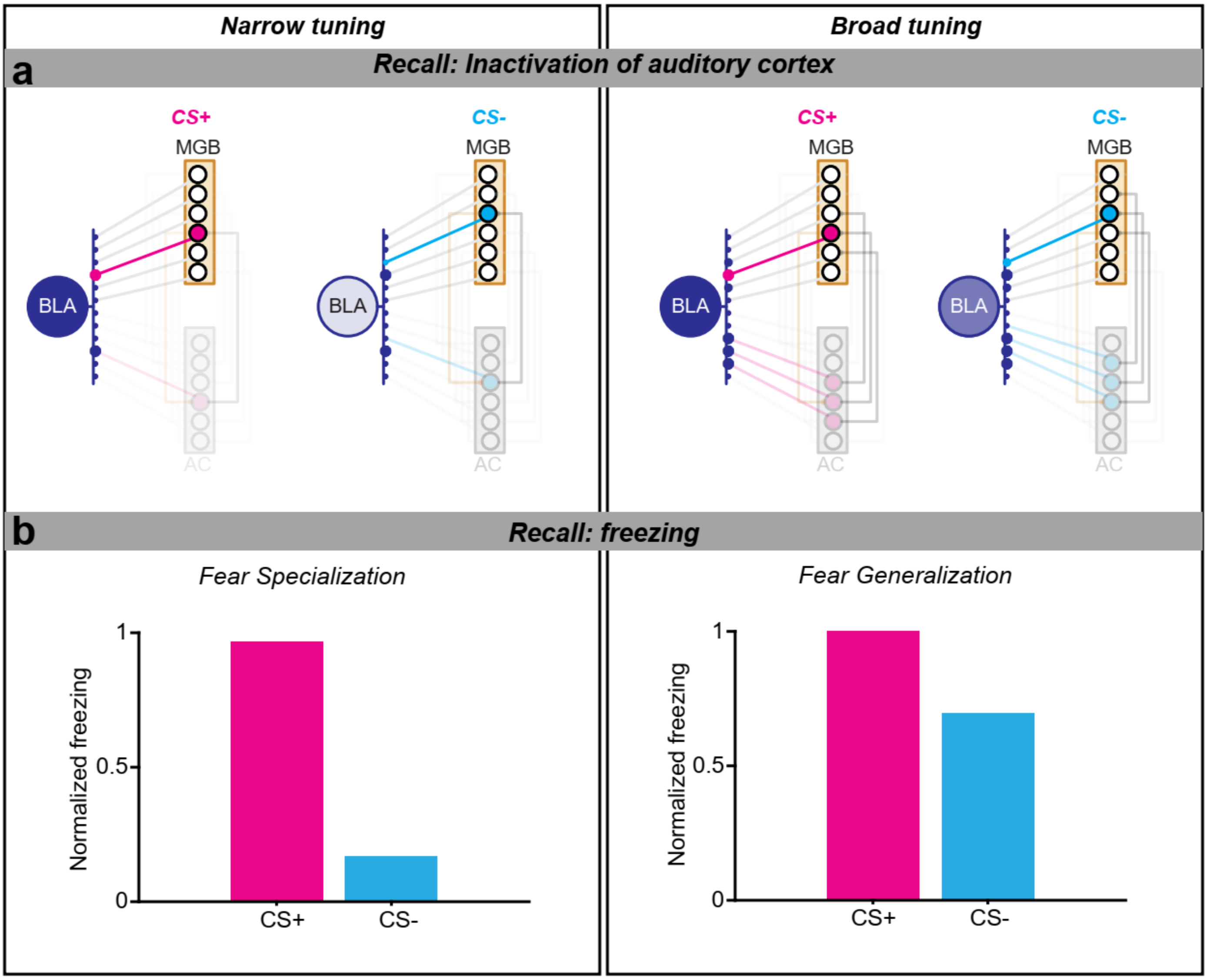

Supplementary Figure 15: Model schematic with AC inactivation during memory recall. Activation and consolidation during DFC are the same as Supplementary Fig. 12 **(a)** shows activation of MBG and BLA without the presence of AC. With broad tuning, CS- still activated strengthened MGB-BLA connections thus increasing BLA activation. **(b)** Results of freezing during memory recall are very similar to when there are no interventions (Supplementary Fig. 12). Source data are provided as a Source Data file.

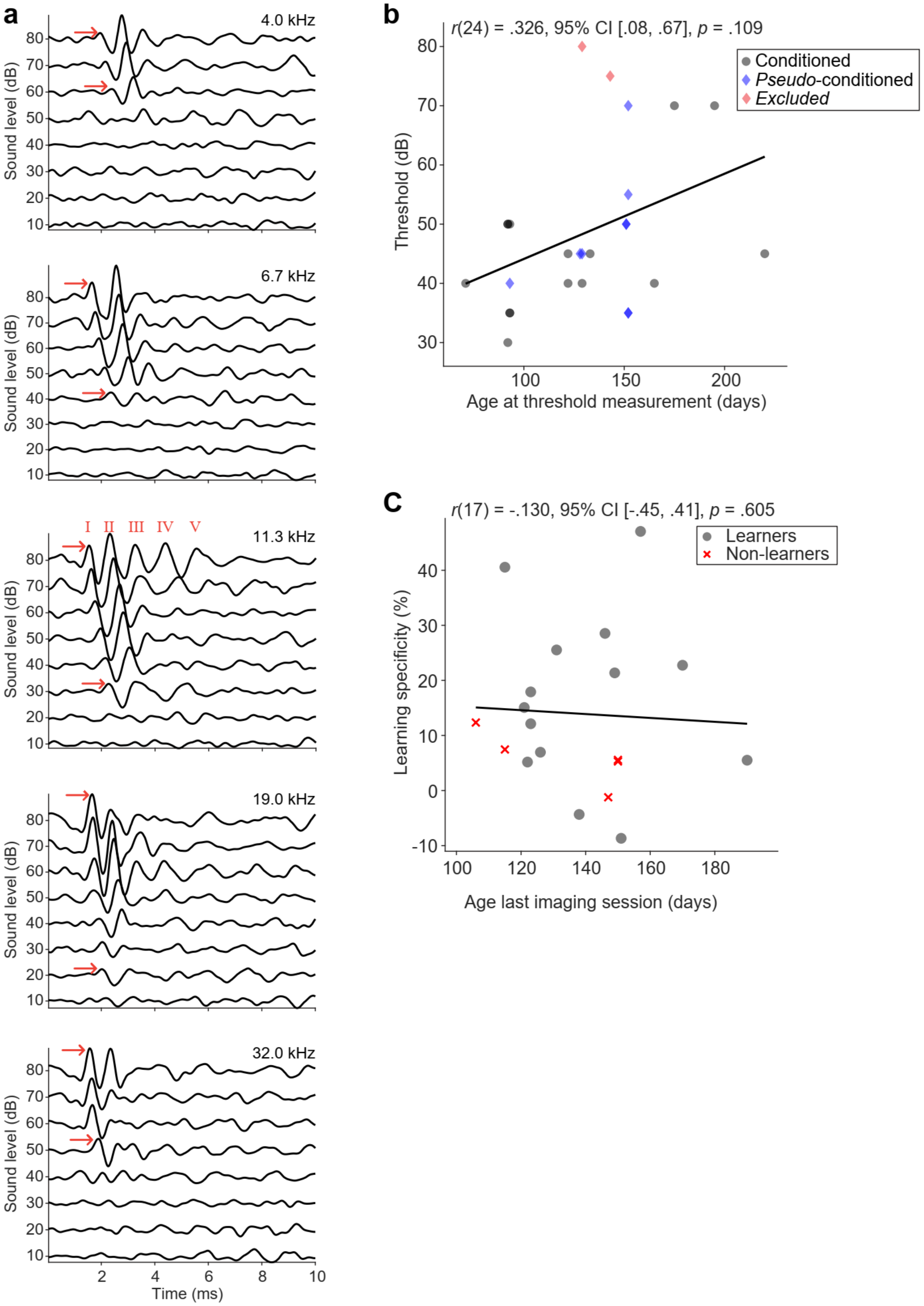

Supplementary Figure 16: Auditory Brainstem Responses. (a) Example ABR responses to 5 frequencies presented at 7 different sound levels (10 – 80 dB SPL). Red arrows indicate Wave I at the highest and lowest detected threshold. Waves are indicated in red (I – V) in the 11.3 kHz panel. **(b)** Relationship between age at ABR threshold measurement and threshold (mean of frequencies closest to CS+ and CS-). Mice with thresholds greater than 70dB (red) were excluded from the study. **(c)** Relationship between age at the last imaging session and learning specificity. Black lines in **b** and **c** are best linear fits. Statistics **b** & **c**: two-tailed Spearman's rank correlation. Source data are provided as a Source Data file.

Figure	Comparison	N	Test Results
1c	Two-way ANOVA: Effect of cond_type (conditioned [c], pseudo-conditioned [pc]) and stimulus (CS-, CS+, baseline [bl]) on freezing (%)	23 mice	see below

Sum of squares:	*F-statistic (DF)*	*p-value*
cond_type = 472.90	F(1,63) = 472.9	0.032
stimulus = 1565.9	F(2,63) = 783.0	0.001
cond_type*stimulus = 2536.6	F(2,63) = 1268.3	< 0.001

post-hoc Tukey-Kramer testing															
Group	pc & bl	pc & bl	pc & bl	pc & bl	pc & bl	c & bl	c & bl	c & bl	c & bl	pc & CS-	pc & CS-	pc & CS-	c & CS-	c & CS-	pc & CS+
	c & bl	pc & CS-	c & CS-	pc & CS+	c & CS+	pc & CS-	c & CS-	pc & CS+	c & CS+	c & CS-	pc & CS+	c & CS+	pc & CS+	c & CS+	c & CS+
lower CI	-2.45	-9.13	-13.48	-10.09	-29.25	-17.83	-22.03	-18.79	-37.80	-18.08	-14.69	-33.84	-7.76	-26.77	-32.88
estimated mean	9.99	4.60	-1.04	3.63	-16.81	-5.39	-11.03	-6.35	-26.79	-5.64	-0.96	-21.40	4.67	-15.77	-20.44
upper CI	22.43	18.32	11.40	17.36	-4.37	7.05	-0.02	6.09	-15.79	6.80	12.76	-8.97	17.11	-4.76	-8.00
p-value	0.186	0.921	1.000	0.970	0.002	0.798	0.049	0.665	0.000	0.766	1.000	0.000	0.878	0.001	0.000

Figure	Comparison	N
1d	Mixed-effects model: freezing ~ 1 + stim_type*session + (1|mouse)	14 mice

Model Coefficients: Estimate ± standard error	*t-statistic (DF)*	*p-value*
Intercept: -12.67 ± 5.07	t(164) = -2.50	0.014
Session: 1.66 ± 1.85	t(164) = 0.90	0.372
stim_type: 15.63 ± 2.35	t(164) = 6.66	< 0.001
session*stim_type: -1.04 ± 0.86	t(164) = -1.21	0.227

Figure	Comparison	N
1e	Mixed-effects model: freezing ~ 1 + stim_type*session + (1|mouse)	9 mice

Model Coefficients: Estimate ± standard error	*t-statistic (DF)*	*p-value*
Intercept: 13.57 ± 3.75	t(104) = 3.62	0.001
Session: 1.26 ± 1.37	t(104) = 0.92	0.359
stim_type: -0.89 ± 1.74	t(104) = -0.51	0.608
session*stim_type: -0.75 ± 0.63	t(104) = -1.19	0.236

Figure	Comparison	N
4a	Two-way repeated measures ANOVA: Effect of time (pre-DFC, post-DFC) and cond_type (conditioned, pseudo-conditioned) on Zdiff	23 mice

Sum of squares:	*F-statistic (DF)*	*p-value*	*post-hoc Tukey-Kramer testing*		
time = 0.002	F(1,21) = 2.65	0.118	Cond_type	DFC	pseudo-DFC
time*cond_type = 0.003	F(1,21) = 4.20	0.053	Time	pre-DFC vs post-DFC	
			p-value	0.740	0.028

Figure	Comparison	N
4b	Two-way repeated measures ANOVA: Effect of time (pre-DFC, post-DFC) and cond_type (conditioned, pseudo-conditioned) on SVM performance	23 mice

Sum of squares:	*F-statistic (DF)*	*p-value*	*post-hoc Tukey-Kramer testing*		
time = 73.43	F(1,21) = 10.88	0.003	Cond_type	DFC	pseudo-DFC
time*cond_type = 45.00	F(1,21) = 6.67	0.017	Time	pre-DFC vs post-DFC	
			p-value	0.573	0.001

Figure	Comparison	N
5a panel 1	Two-way repeated measures ANOVA: Effect of time (pre-DFC, post-DFC) and stimulus (frequency) on response magnitude (ΔF/Fstd)	1 neuron

Sum of squares:	*F-statistic (DF)*	*p-value*	*post-hoc Tukey-Kramer testing*												
time = 6.77	F(1,348) = 13.18	< 0.001	Frequency (kHz)	5.0	6.1	7.6	9.3	11.4	12.5	13.7	15.0	17.2	21.2	26.0	32.0
time*stimulus = 14.15	F(11,348) = 2.5	0.005	Time	pre-DFC vs post-DFC											
			p-value	0.115	0.644	0.342	0.533	0.045	0.012	0.004	0.001	0.007	0.819	0.626	0.397

Figure	Comparison	N
5a panel 2	Two-way repeated measures ANOVA: Effect of time (pre-DFC, post-DFC) and stimulus (frequency) on response magnitude (ΔF/Fstd)	1 neuron

Sum of squares:	*F-statistic (DF)*	*p-value*	*post-hoc Tukey-Kramer testing*												
time = 0.01	F(1,288) = 0.01	0.917	Frequency (kHz)	5.0	6.1	7.6	9.3	11.4	12.5	13.7	15.0	17.2	21.2	26.0	32.0
time*stimulus = 17.76	F(11,248) = 2.55	0.004	Time	pre-DFC vs post-DFC											
			p-value	0.613	0.557	0.940	0.160	0.002	0.378	0.131	0.002	0.174	0.721	0.524	0.429

Figure	Comparison	N
5a panel 3	Two-way repeated measures ANOVA: Effect of time (pre-DFC, post-DFC) and stimulus (frequency) on response magnitude (ΔF/Fstd)	1 neuron

Sum of squares:	*F-statistic (DF)*	*p-value*	*post-hoc Tukey-Kramer testing*												
time = 188.02	F(1,288) = 11.74	0.001	Frequency (kHz)	5.0	6.1	7.6	9.3	11.4	12.5	13.7	15.0	17.2	21.2	26.0	32.0
time*stimulus = 1194.00	F(11,288) = 6.78	< 0.001	Time	pre-DFC vs post-DFC											
			p-value	0.968	0.937	0.945	0.000	0.237	0.457	0.000	0.861	0.348	0.959	0.431	0.694

Figure	Comparison	N
5b	Two-way repeated measures ANOVA: Effect of time (pre-DFC, post-DFC) and stimulus (frequency) on response magnitude (ΔF/Fstd)	879 neurons

Sum of squares:	*F-statistic (DF)*	*p-value*	*post-hoc Tukey-Kramer testing*												
time = 0.81	F(1,10536) = 11.99	0.001	Frequency (kHz)	5.0	6.1	7.6	9.3	11.4	12.5	13.7	15.0	17.2	21.2	26.0	32.0
time*stimulus = 3.01	F(11,10536) = 4.06	< 0.001	Time	pre-DFC vs post-DFC											
			p-value	0.377	0.232	0.147	0.062	0.309	0.004	0.002	< 0.001	0.002	0.786	0.294	0.695

Figure	Comparison	N
5c	Two-way repeated measures ANOVA: Effect of time (pre-DFC, post-DFC) and stimulus (frequency) on response magnitude (ΔF/Fstd)	626 neurons

Sum of squares:	*F-statistic (DF)*	*p-value*	*post-hoc Tukey-Kramer testing*												
time = 0.52	F(1,7500) = 8.07	0.005	Frequency (kHz)	5.0	6.1	7.6	9.3	11.4	12.5	13.7	15.0	17.2	21.2	26.0	32.0
time*stimulus = 1.52	F(11,7500) = 2.16	0.014	Time	pre-DFC vs post-DFC											
			p-value	< 0.001	0.103	0.449	0.687	0.967	0.635	0.184	0.177	0.786	0.401	0.939	0.014

Figure	Comparison	N
6c	Two-way repeated measures ANOVA: Effect of time (pre-DFC, post-DFC) and cond_type (conditioned, pseudo-conditioned) on distance of BF from CS+	1505 neurons

Sum of squares:	*F-statistic (DF)*	*p-value*	*post-hoc Tukey-Kramer testing*		
time = 0.86	F(1,1503) = 108.78	0.026	Cond_type	DFC	pseudo-DFC
time*cond_type = 0.96	F(1,1503) = 1.38	0.018	Time	pre-DFC vs post-DFC	
			p-value	< 0.001	0.934

Figure	Comparison	N
6d	Two-way repeated measures ANOVA: Effect of time (pre-DFC, post-DFC) and cond_type (conditioned, pseudo-conditioned) on sparseness	1505 neurons

Sum of squares:	*F-statistic (DF)*	*p-value*	*post-hoc Tukey-Kramer testing*		
time = 0.23	F(1,1503) = 20.93	< 0.001	Cond_type	DFC	pseudo-DFC
time*cond_type = 0.002	F(1,1503) = 0.21	0.649	Time	pre-DFC vs post-DFC	
			p-value	0.001	0.001

Figure	Comparison	N
S3b-c	Mixed-effects model: learning_specificity ~ 1 + controls*session + (1 | mouse)	23 mice

Model Coefficients: Estimate ± standard error	*t-statistic (DF)*	*p-value*
Intercept: 16.19 ± 4.24	t(88) = 3.82	< 0.001
conditioning_type: -14.95 ± 6.78	t(88) = -2.20	0.030
session: 0.26 ± 1.10	t(88) = 0.23	0.817
session*conditioning_type: -0.01 ± 1.76	t(88 = -0.01)	0.995

Figure	Comparison	N
S5f	Kruskalwallis: Effect of best frequency on Zdiff	2185 neurons

Sum of squares:	*H-statistic (DF)*	*p-value*
best frequency = 6.45 e+07	X^2(11,2173) = 5.86 e+06	< 0.001

post-hoc Tukey-Kramer testing

Best freq. (kHz)	5.0	5.0	5.0	5.0	5.0	5.0	5.0	5.0	5.0	5.0	5.0	6.1	6.1	6.1	6.1	6.1	6.1	6.1	6.1	6.1	6.1	7.6
	6.1	7.6	9.3	11.4	12.5	13.7	15.0	17.2	21.2	26.0	32.0	7.6	9.3	11.4	12.5	13.7	15.0	17.2	21.2	26.0	32.0	9.3
lower CI	-105.2	-170.6	-227.8	-754.8	-152.5	-241.0	-614.1	-275.2	-149.0	-163.0	-116.4	-267.7	-325.3	-850.9	-248.4	-336.9	-710.4	-371.6	-246.4	-260.0	-214.5	-272.8
estimated mean	85.5	26.6	-37.9	-538.9	68.9	-19.6	-401.2	-63.3	43.0	37.5	63.8	-58.8	-123.4	-624.3	-16.6	-105.1	-486.6	-148.8	-42.5	-47.9	-21.7	-64.6
upper CI	276.1	223.9	151.9	-323.0	290.3	201.8	-188.3	148.6	235.0	238.1	244.0	150.1	78.5	-397.8	215.2	126.8	-262.9	74.1	161.5	164.1	171.2	143.6
p-value	0.950	1.000	1.000	0.000	0.997	1.000	0.000	0.998	1.000	1.000	0.992	0.999	0.696	0.000	1.000	0.946	0.000	0.563	1.000	1.000	1.000	0.997

Best freq. (kHz)	7.6	7.6	7.6	7.6	7.6	7.6	7.6	7.6	9.3	9.3	9.3	9.3	9.3	9.3	9.3	9.3	11.4	11.4	11.4	11.4	11.4	11.4
	11.4	12.5	13.7	15.0	17.2	21.2	26.0	32.0	11.4	12.5	13.7	15.0	17.2	21.2	26.0	32.0	12.5	13.7	15.0	17.2	21.2	26.0
lower CI	-797.7	-195.1	-283.5	-657.2	-318.4	-193.7	-207.1	-162.2	-726.9	-124.4	-212.9	-586.3	-247.5	-122.2	-135.9	-90.4	354.7	266.3	-107.9	230.8	354.2	341.4
estimated mean	-565.5	42.2	-46.2	-427.8	-89.9	16.4	10.9	37.2	-500.9	106.8	18.3	-363.2	-25.4	81.0	75.5	101.8	607.7	519.3	137.7	475.6	581.9	576.4
upper CI	-333.3	279.5	191.1	-198.4	138.6	226.5	228.9	236.6	-275.0	338.0	249.5	-140.2	196.8	284.1	286.8	293.9	860.8	772.3	383.3	720.4	809.6	811.4
p-value	0.000	1.000	1.000	0.000	0.981	1.000	1.000	1.000	0.000	0.938	1.000	0.000	1.000	0.979	0.991	0.854	0.000	0.000	0.800	0.000	0.000	0.000

Best freq. (kHz)	11.4	12.5	12.5	12.5	12.5	12.5	12.5	13.7	13.7	13.7	13.7	13.7	15.0	15.0	15.0	15.0	17.2	17.2	17.2	21.2	21.2	26.0
	32.0	13.7	15.0	17.2	21.2	26.0	32.0	15.0	17.2	21.2	26.0	32.0	17.2	21.2	26.0	32.0	21.2	26.0	32.0	26.0	32.0	32.0
lower CI	384.8	-346.2	-720.5	-381.8	-258.8	-271.4	-228.4	-632.0	-293.4	-170.3	-182.9	-139.9	95.7	219.3	206.4	250.1	-117.7	-130.6	-86.9	-218.7	-173.4	-176.5
estimated mean	602.7	-88.5	-470.0	-132.2	-25.9	-31.3	-5.1	-381.6	-43.7	62.6	57.1	83.4	337.9	444.2	438.7	465.0	106.3	100.8	127.1	-5.5	20.8	26.3
upper CI	820.6	169.3	-219.6	117.5	207.1	208.8	218.3	-131.1	206.0	295.5	297.2	306.8	580.0	669.1	671.0	679.9	330.3	332.2	341.1	207.8	215.0	229.0
p-value	0.000	0.994	0.000	0.855	1.000	1.000	1.000	0.000	1.000	0.999	1.000	0.987	0.000	0.000	0.000	0.000	0.926	0.959	0.733	1.000	1.000	1.000

Figure	Comparison	N
S6b	linear model: ΔSVM ~ 1 + days_apart*conditioning_type	28 observations

Model Coefficients: Estimate ± standard error	*t-statistic (DF)*	*p-value*
Intercept: -2.16 ± 1.27	t(52) = -1.70	0.094
days_apart: -0.19 ± 0.37	t(52) = -0.53	0.596
conditioning_type: -0.17 ± 1.79	t(52) = -0.10	0.925
days_apart*conditioning_type: -1.04 ± 0.86	t(52) = -2.39	0.020

Figure	Comparison	N
S6d	linear model: ΔZdiff ~ 1 + days_apart*conditioning_type	28 observations

Model Coefficients: Estimate ± standard error	*t-statistic (DF)*	*p-value*
Intercept: -0.01 ± 0.01	t(52) = -0.76	0.449
days_apart: 0.0001 ± 0.0038	t(52) = 0.04	0.965
conditioning_type: 0.03 ± 0.02	t(52) = 1.68	0.100
days_apart*conditioning_type: -0.02 ± 0.005	t(52) = -3.00	0.004

Figure	Comparison	N
S6e	Mixed-effects model: similarity ~ 1 + days_apart*cond_type + (1 | mouse)	28 observations

Model Coefficients: Estimate ± standard error	*t-statistic (DF)*	*p-value*
Intercept: 0.42 ± 0.06	t(624) = 7.10	< 0.001
days_apart: -0.03 ± 0.01	t(624) = -2.87	0.004
cond_type: -0.25 ± 0.10	t(624) = -2.64	0.009
days_apart*cond_type: 0.02 ± 0.02	t(624) = 1.28	0.201

Figure	Comparison	N
S7b	Two-way repeated measures ANOVA: Effect of time (pre-DFC, post-DFC) and stimulus (frequency) on response magnitude (ΔF/Fstd)	879 neurons

Sum of squares:	*F-statistic (DF)*	*p-value*	*post-hoc Tukey-Kramer testing*												
time = 0.94	F(1,10536) = 16.24	< 0.001	Frequency (kHz)	5.0	6.1	7.6	9.3	11.4	12.5	13.7	15.0	17.2	21.2	26.0	32.0
time*stimulus = 1.13	F(11,10536) = 1.78	0.051	Time	pre-DFC vs post-DFC											
			p-value	0.799	0.016	0.061	0.001	0.112	0.790	0.717	0.211	0.851	0.213	0.115	0.008

Figure	Comparison	N
S7c	Two-way repeated measures ANOVA: Effect of time (pre-DFC, post-DFC) and stimulus (frequency) on response magnitude (ΔF/Fstd)	626 neurons

Sum of squares:	*F-statistic (DF)*	*p-value*	*post-hoc Tukey-Kramer testing*												
time = 12.27	F(1,7500) = 108.78	< 0.001	Frequency (kHz)	5.0	6.1	7.6	9.3	11.4	12.5	13.7	15.0	17.2	21.2	26.0	32.0
time*stimulus = 1.71	F(11,7500) = 1.38	0.174	Time	pre-DFC vs post-DFC											
			p-value	0.980	0.004	0.026	< 0.001	< 0.001	0.035	0.000	0.005	< 0.001	< 0.001	< 0.001	0.004

Figure	Comparison	N
S7e (top 20th percentile)	Two-way ANOVA: Effect of time (pre-DFC, post-DFC) and stimulus (CS+/CS-) on response magnitude (ΔF/Fstd)	704 neurons

Sum of squares:	*F-statistic (DF)*	*p-value*	*post-hoc Tukey-Kramer testing*						
time = 0.12	F(1,700) = 1.21	0.106	stimulus*time	CS-, pre	CS-, pre	CS-, pre	CS+, pre	CS+, pre	CS-, post
stimulus = 0.26	F(1,700) = 2.62	0.272	stimulus*time	CS+, pre	CS-, post	CS+, post	CS-, post	CS+, post	CS+, post
time*stimulus = 0.12	F(1,700) = 1.23	0.267	*p-value*	0.215	0.400	0.218	0.983	1.000	0.984

Figure	Comparison	N
S7e (bottom 20th percentile)	Two-way ANOVA: Effect of time (pre-DFC, post-DFC) and stimulus (CS+/CS-) on response magnitude (ΔF/Fstd)	704 neurons

Sum of squares:	*F-statistic (DF)*	*p-value*	*post-hoc Tukey-Kramer testing*						
time = 0.17	F(1,700) = 19.26	< 0.001	stimulus*time	CS-, pre	CS-, pre	CS-, pre	CS+, pre	CS+, pre	CS-, post
stimulus = 0.23	F(1,700) = 26.79	< 0.001	stimulus*time	CS+, pre	CS-, post	CS+, post	CS-, post	CS+, post	CS+, post
time*stimulus = 0.02	F(1,700) = 2.23	0.136	*p-value*	0.046	0.171	< 0.001	0.945	< 0.001	< 0.001

Figure	Comparison	N
S7g (top 20th percentile)	Two-way ANOVA: Effect of time (pre-DFC, post-DFC) and stimulus (CS+/CS-) on response magnitude (ΔF/Fstd)	504 neurons

Sum of squares:	*F-statistic (DF)*	*p-value*	*post-hoc Tukey-Kramer testing*						
time = 1.91	F(1,500) = 8.16	0.004	stimulus*time	CS-, pre	CS-, pre	CS-, pre	CS+, pre	CS+, pre	CS-, post
stimulus = 2.01	F(1,500) = 8.61	0.005	stimulus*time	CS+, pre	CS-, post	CS+, post	CS-, post	CS+, post	CS+, post
time*stimulus = 0.03	F(1,500) = 0.11	0.745	*p-value*	0.097	0.110	< 0.001	1.000	0.278	0.252

Figure	Comparison	N
S7g (bottom 20th percentile)	Two-way ANOVA: Effect of time (pre-DFC, post-DFC) and stimulus (CS+/CS-) on response magnitude (ΔF/Fstd)	504 neurons

Sum of squares:	*F-statistic (DF)*	*p-value*	*post-hoc Tukey-Kramer testing*						
time = 0.001	F(1,500) = 0.14	0.068	stimulus*time	CS-, pre	CS-, pre	CS-, pre	CS+, pre	CS+, pre	CS-, post
stimulus = 0.03	F(1,500) = 0.07	0.711	stimulus*time	CS+, pre	CS-, post	CS+, post	CS-, post	CS+, post	CS+, post
time*stimulus = 0.002	F(1,500) = 0.23	0.635	*p-value*	0.775	1.000	0.405492	0.732	0.933	0.363

Figure	Comparison	N
S11e	Two-way repeated measures ANOVA: Effect of time (pre-DFC, post-DFC) and putative auditory cortex location (A1, AAF) on Zdiff	14 mice

Sum of squares:	*F-statistic (DF)*	*p-value*	*post-hoc Tukey-Kramer testing*		
time = 0.00004	F(1,12) = 0.05	0.825	Location	A1	AAF
time*cond_type = 0.0002	F(1,12) = 0.30	0.597	Time	pre-DFC vs post-DFC	
			p-value	0.568	0.837

Supplementary Table 1: Statistics for all figures.

Source	Sum sq.	degrees of freedom	Mean sq.	F	p-value
Intercept:Time	1.02	1	1.02	15.59	0.000
stimulus:Time	0.62	2	0.31	4.71	0.009
Error(Time)	237.18	3625	0.07		

post-hoc Tukey-Kramer testing

Stimulus	Time		Difference	Std. Error	p-value	lower confidence interval	upper confidence interval
CS+	pre-DFC	post-DFC	-0.05	0.01	0.00001	-0.08	-0.03
CS-	pre-DFC	post-DFC	-0.01	0.01	0.302	-0.04	0.01
CSc	pre-DFC	post-DFC	-0.01	0.01	0.251	-0.03	0.01

Supplementary Table 2: Statistics comparing normalized responses at CS+, CS- and CSc. (Upper panel) Two-way repeated measures ANOVA with dependent variable mean response (across cells) and independent variables; stimulus (CS+, CS-, CSc) and Time (pre-DFC, post-DFC). (Lower panel) post-hoc Tukey-Kramer testing.

Source	Sum sq.	degrees of freedom	Mean sq.	F	p-value
Intercept:Time	0.67	1	0.67	8.30	0.004
stimulus:Time	1.62	2	0.81	10.10	0.000
Error(Time)	291.01	3625	0.08		

post-hoc Tukey-Kramer testing

Stimulus	Time		Difference	Std. Error	p-value	lower confidence interval	upper confidence interval
CS+	pre-DFC	post-DFC	-0.01	0.01	0.289	-0.04	0.01
CS-	pre-DFC	post-DFC	0.02	0.01	0.178	-0.01	0.04
CSc	pre-DFC	post-DFC	0.06	0.01	0.000	0.04	0.08

Supplementary Table 3: Statistics comparing non-normalized responses at CS+, CS- and CSc. (Upper panel) Two-way repeated measures ANOVA with dependent variable mean normalized response (across cells) and independent variables; stimulus (CS+, CS-, CSc) and Time (pre-DFC, post-DFC). (Lower panel) post-hoc Tukey-Kramer testing.

Supplementary References

1. Jonas. (2021) Violin Plots for plotting multiple distributions (distributionPlot.m) (https://www.mathworks.com/matlabcentral/fileexchange/23661-violin-plots-for-plotting-multiple-distributions-distributionplot-m), MATLAB Central File Exchange. Retrieved August 2. (2021).

2. Franklin, K. & Paxinos, G. *The mouse brain in stereotaxic coordinates 3rd edition*. (Academic Press, 2007).

3. Romero, S. *et al.* Cellular and Widefield Imaging of Sound Frequency Organization in Primary and Higher Order Fields of the Mouse Auditory Cortex. *Cereb. Cortex* **30**, 1603–1622 (2020).
